# Study on Properties of Automatically Designed 3D-Printed Customized Prosthetic Sockets

**DOI:** 10.3390/ma14185240

**Published:** 2021-09-12

**Authors:** Filip Górski, Radosław Wichniarek, Wiesław Kuczko, Magdalena Żukowska

**Affiliations:** Faculty of Mechanical Engineering, Poznan University of Technology, Piotrowo 3 STR, 61-138 Poznan, Poland; radoslaw.wichniarek@put.poznan.pl (R.W.); wieslaw.kuczko@put.poznan.pl (W.K.); magdalena.zukowska@put.poznan.pl (M.Ż.)

**Keywords:** additive manufacturing, mechanical properties, medical 3D printing, prosthetic sockets, customization

## Abstract

This paper presents the results of experiments conducted on a batch of additively manufactured customized prosthetic sockets for upper limbs, made of thermoplastics and designed automatically on the basis of a 3D-scanned limb of a 3-year-old patient. The aim of this work was to compare sockets made of two different materials—rigid PLA and elastic TPE. Two distinct socket designs with various mounting systems were prepared. To find a reliable set of parameters for cheap and stable manufacturing of usable prostheses using 3D printers, realizing the fused deposition modeling (FDM) process, sets of sockets were manufactured with various process parameters. This paper presents the methodology of the design, the plan of the experiments and the obtained results in terms of process stability, fit and assessment by patient, as well as strength of the obtained sockets and their measured surface roughness. The results are promising, as most of the obtained products fulfil the strength criteria, although not all of them meet the fitting and use comfort criteria. As a result, recommendations of materials and process parameters were determined. These parameters were included in a prototype of the automated design and production system developed by the authors, and prostheses for several other patients were manufactured.

## 1. Introduction

One of the consequences of the SARS-CoV-2 virus and the emerging problems concerning the lack of resources, especially for personal protection, but also medical machine components [1], was a significant increase in the awareness of the medical community about the possibilities of additive manufacturing technology [2]. In the mainstream media, it is most often called “3D printing”, which significantly facilitates the visual presentation and understanding of the basics of this technology for people without a technical background. In technical areas, additive manufacturing has been successfully used for many years, not only for the production of prototypes [3], but also of final products or their parts [4]. The scientific literature is also full of examples of attempts to implement additive manufacturing methods in medicine. The common feature of the vast majority of these examples is the uniqueness of the geometry produced, tailored to the needs of a particular patient or medic. In general, the medical applications of additively produced elements can be divided into several areas: bioprinting (tissue and organ production) [5], prosthetic and orthopedic equipment [6], teaching aids, pre- and intraoperative supplies [7] and implants [8].

The main aim of the research work described in the paper is the design and realization of experiments focused on the selection of materials, geometry and parameters of an additive manufacturing process for modular individualized upper limb prostheses, designed automatically, on the basis of a 3D scan of a given patient. The system is aimed mostly at child patients—the obtained prostheses are supposed to be cheap and quickly replaceable as soon as the patient’s limb grows out and does not fit the currently used prosthesis. In view of the authors’ experience, in cases of children under the age of 10, this is an interval which is almost never longer than one year (e.g., a 4-year-old child will not be able to use a customized prosthesis made for them at the age of three). The following parts of the paper present relevant literature, the methodology of designing and testing the individualized prosthetic sockets and the results of these tests.

## 2. Literature Review

### 2.1. FDM Technology Overview

Decades of development of additive manufacturing technology have resulted in the creation of many different production methods, in which an element is produced layer by layer directly from a digital model. One of the most widespread methods of additive manufacturing is fused deposition modeling (FDM) technology. The success of its range is due, among other things, to its low purchase price, which makes it available not only to industrial users. In the latter case, it is more commonly referred to as fused filament fabrication (FFF). The wide range of thermoplastic materials that can be processed with this method means that it finds many practical applications [9]. Polylactic acid (PLA) is one of the materials that is used frequently in the medical industry, including in soluble threads. Processed with the FDM method, it retains good strength properties, and thanks to its organic origin and biodegradability, it is considered environmentally friendly [10]. In the FDM process, it is possible to use several different materials at the same time, thanks to which, within one product, it is possible to combine hard PLA material and much more flexible and pleasant-to-the-touch thermoplastic polyester (TPE) [11].

### 2.2. Problems of Traditional Prosthetic Socket Manufacturing

**Figure 1:**
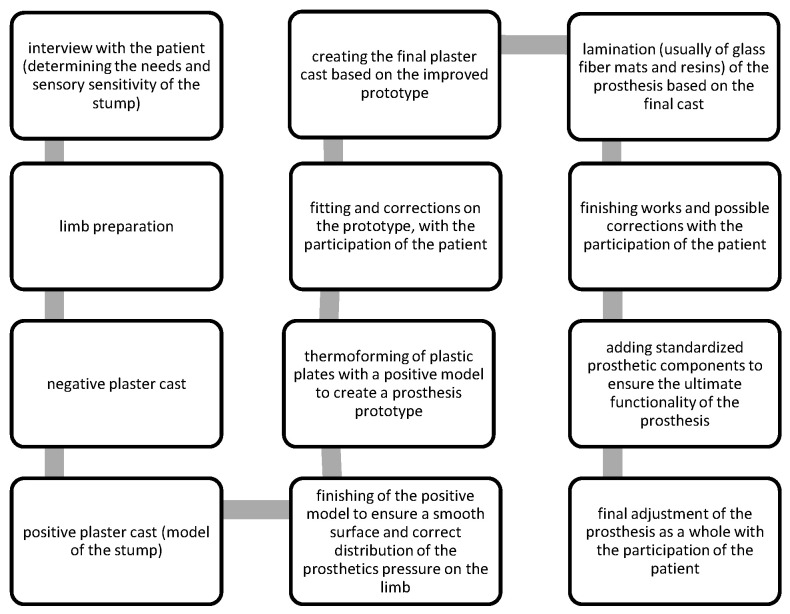
Flow graph—traditional manufacturing of prosthetic sockets, adapted from Ref. [13].

In order to utilize 3D printing as a method of production of prosthetics, one must first prepare a 3D digital model of a prosthesis for a given patient. This is not a frequent case in the currently prevailing model of prosthesis production. It should be noted that descriptions of some of the first attempts to introduce computerized systems of designing and manufacturing prostheses can be found in the literature from almost thirty years ago [12]. However, most of the prostheses available to the average recipient are still made using traditional prosthetic technologies. This is especially true regarding prosthetic sockets. This part of the prosthesis must be perfectly adapted to the patient’s anatomy. The fabrication processes may vary slightly depending on the particular manufacturer and available tools. However, there is a list of steps necessary to produce the prosthesis in a conventional manner [13], as shown in Figure 1.

In the authors’ opinion (confirmed by interviews with patients, doctors and physiotherapists made prior to the beginning of the research), this method of production has three main disadvantages. The first one concerns the production time of the prosthesis. Apart from the issues of available production capacity, it may take several days to produce the simplest complete prosthesis, from scratch, under favorable conditions. Professional body-powered prosthetics may need months to be produced. Moreover, this process requires many hours of patient involvement (in multiple sessions), which can often be a logistical challenge for the patient (especially if pediatric patients are concerned). This translates into a significant total cost of the prosthesis for the end user. The last relative disadvantage is the significant influence of the prosthetist’s manual skills on the final quality of the product. Traditional production of prostheses more often resembles handcraft than the industrial production of the 21st century. In developed countries, there is access to the necessary production workshop and appropriately qualified staff, and there are also opportunities to train new employees. Unfortunately, in less developed countries it is not possible to easily increase the availability of prostheses [14].

The problem of accessibility of limb prostheses for children is even greater than in the case of adults. This is due to the fact that changes in human anatomy (i.e., limb dimensions) rapidly progress during adolescence, while the design of prostheses does not allow for their smooth adjustment as their users grow. This means a much more frequent need to change prostheses (similarly to clothes or shoes, which are rarely used for longer than one year by children aged less than 10), which, on the one hand, are not yet worn out, and, on the other hand, cannot be used by another patient to the fullest extent, due to the individual nature of the product [15]. Children growing up with upper limb deficiencies are weak in motor skills. These problems worsen with the age of the child [16]. Appropriate therapy, combined with the use of appropriate and adapted prosthetic equipment, allows problems with incorrect motor skills and motor development of a young person to be alleviated [17].

### 2.3. Research Problem Identification

The use of the FDM method in the production of prosthetic products, in combination with the three-dimensional scanning technique, was aimed at eliminating the previously indicated disadvantages of traditional manufacturing methods. The main advantages of modern prostheses can therefore be described as follows:

- Thanks to the digital documentation of the patient’s limb, their presence in the product design and production process can be limited to a minimum [18]; in some situations, the measurement of a patient’s limb may even be done completely remotely [19].
- Products manufactured using the FDM method can be more individualized [20].
- Additive manufacturing of individualized prosthetic devices takes less time [21].
- The cost of production is lower, which improves the availability of prosthetic devices [13]; they can therefore be changed more often in fast-growing children.
- The production process does not require high engineering nor technical skills from the worker, apart from basic computer skills [22].

However, despite the advantages of using the FDM method in prosthetics indicated in the available literature, the implementation of this modern manufacturing method in industrial practice progresses slower than expected [23]. The problem of testing the strength of additive-manufactured products, especially in the case of their individualized nature, is an important issue. As the examples described in the literature show, there have been accidents involving physical damage to a medical device during tests carried out directly on patients [24]. These products were made with additive methods other than FDM, in theory guaranteeing greater material strength than FDM. For the latter technology, a significant problem is also the large anisotropy of mechanical properties, which makes it difficult to design products with complex shapes and loads [25]. In the case of products intended to protect human health, in order to obtain the necessary strength it may be necessary to optimize the topology of the product due to its strength, but at the expense of weight [26]. In the optimization of the structure (e.g., wall thickness) it is possible to use appropriate numerical calculations. However, due to the complicated shape of some products and the complex stress system, the calculations necessary for each personalized product may prove to be an uneconomical approach for mass adaptation and will require simplifications [27].

The authors of [21] collected and described 58 examples of various types of upper-limb prostheses, which were wholly or partially produced by additive manufacturing. The described products were the result of both professional research and projects made by hobbyists or non-government organizations (foundations). The authors noticed that the common feature of the described products is a very modest amount of information about the strength properties of prostheses, as well as evidence of the way the prostheses function in the long term. Other researchers note that there are no examples in the literature describing additively manufactured upper-limb prostheses that could be used for recreational or sport purposes [28]. For this type of application, the requirements for both strength and the ability to reproduce the movements of a healthy limb are greater than in the case of general-purpose prostheses.

In the case of all additive-manufactured medical devices, the problem most frequently indicated by the authors is the lack of availability of clear guidelines on procedures related to the safety of the manufacturing and use of these devices [15]. With no routine objective method for the mechanical testing of 3D-printed medical devices, most of the literature examples have their own specific setups [29]. In the absence of a specific standard indicating, for example, the method of measuring the roughness of the prosthetic socket, only universal standards indicating the general method of measuring roughness (EN ISO 4287 and EN ISO 4288) may be used. On the one hand, they are more difficult to reproduce, but in the authors’ opinion, they give a better picture of the situation than testing only classic samples of materials or product sections.

The authors, having carefully analyzed the literature and state of the art, have decided to develop a methodology of automated design and manufacturing of low-cost prosthetic devices for upper limbs in pediatric patients by the use of popular FDM (FFF) technology. This paper presents the results of the manufacturing and tests performed on a batch of additively manufactured prosthetic sockets, meant to be used in cosmetic or mechanical prostheses for children. They were first designed on the basis of a 3D scan of a selected patient, then manufactured using various materials and strategies, and then subjected to both destructive and non-destructive testing. The following part of the paper presents these studies in greater detail.

## 3. Materials and Methods

### 3.1. The AutoMedPrint System

**Figure 2:**
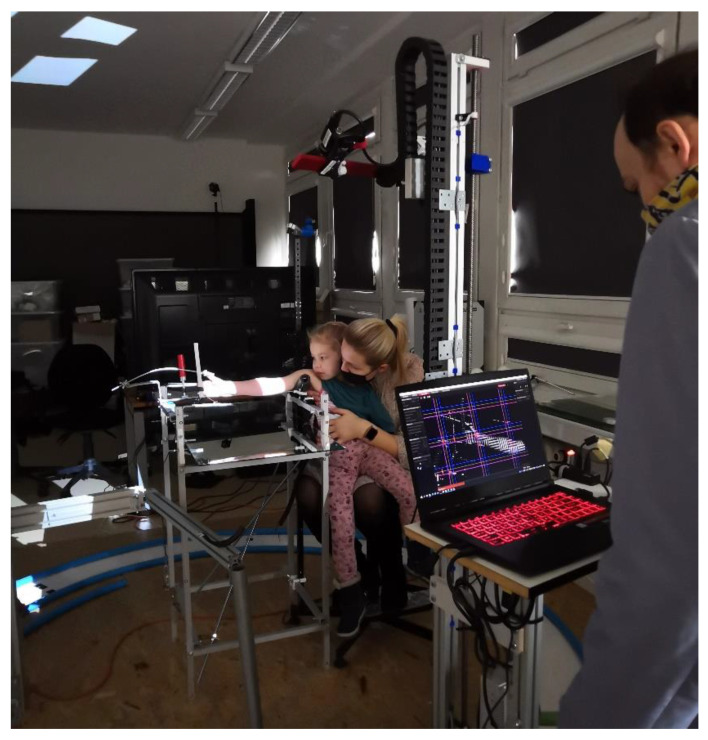
Use of the AutoMedPrint system—3D scanning of a patient.

**Figure 3:**
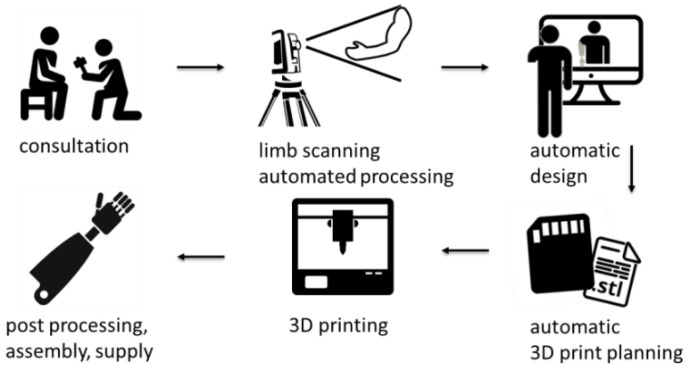
Workflow of the AutoMedPrint system.

The research was realized in the scope of the project “Automation of design and rapid manufacturing of individualized orthopedic and prosthetic supplies on the basis of data of anthropometric measurement”. To solve the problems of existing approaches to the manufacturing of customized orthopedic and prosthetic equipment, an entirely new concept of an automated system is proposed. The system, abbreviated AutoMedPrint (automated 3D printing of medical products), has a task of automated design and production preparation of individualized orthopedic supplies—mainly limb orthoses and upper-limb prostheses. The system’s concept is described in the authors’ earlier works [30,31], and its use is presented in Figure 2, while a flow graph of working with the system is presented in Figure 3.

### 3.2. Design of Customized Prosthetic Sockets

The sockets were designed in an automated way, using existing intelligent CAD models in the Autodesk Inventor software (version 2020, manufactured by the Autodesk company, San Rafael, CA, USA). In general, an intelligent CAD model can be regarded as one that is automatically updated on the basis of knowledge description stored in an external file (a so-called design table) [32]; such models are frequently used for mass customization purposes. The intelligent model was made by the authors, implementing methodologies shown in previous studies [30,31,33] and also using the approach presented in [34]. The complete prosthesis is a modular one, intended for use by children in daily situations; the hand could be either mechanical (a rotary grip for driving a bike or a scooter) or cosmetic (prosthetic hand). As a patient, a 3-year-old girl was selected, with a birth defect of a left upper limb—no elbow joint is present and the arm’s stump is approximately half the length of a healthy arm (measured from shoulder to elbow).

First, the patient’s right arm was 3D scanned on a specially designed work stand, equipped with a David SLS-3 optical scanner (David Vision Systems GmbH, Koblenz, Germany) (visible in Figure 2) moving along a circular track. In total, 6 scans were made—4 direct scans in different positions of the scanner and 2 scans of its mirror image. During the whole process (lasting approx. 5 min), the patient’s forearm rested on a special construction (visible in Figure 2) and remained still. Position of the scanned limb was determined as anatomically correct from a viewpoint of prosthesis use, in accordance with physiotherapists and orthopedists involved as consultants during the design of the stand. Using open-source MeshLab software (version 2019, manufactured by Istituto di Scienza e Tecnologie dell’Informazione, Pisa, Italy), an automated algorithm to process 3D scanning data, created by the authors, was utilized to create a complete mesh of the patient’s limb. The limb was measured digitally in MeshLab to obtain basic lengths and circumferences needed to create a full model of the prosthesis.

**Figure 4:**
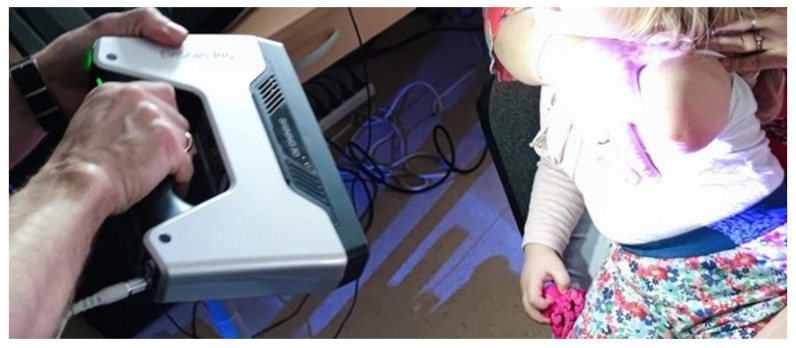
Manual 3D scanning of the patient’s stump.

Then, the stump was 3D scanned using a manual scanner—EinScan 3D Pro (Shining 3D, Hangzhou, China) (Figure 4). The stump was put in a rest, muscles were not flexed, and the proper position was ensured with aid of another, assisting person (a parent in the described case) to ensure gathering of the proper shape of the limb, as advised by medical professionals involved in the system construction and the results of previous preliminary studies on various subjects. Data from the manual scanning were processed in a different way—the recreated mesh of a stump was first positioned in the center of the coordinate system and then subjected to an automated algorithm of section creation in MeshLab (using a custom-made macro, executing a number of compute planar section filters). Six section planes were made perpendicularly to the arm axis and each were 14 mm—they were based on the total length of the stump, measured manually in MeshLab. Each section in MeshLab contains a set of points. The point coordinates were exported to a text file, and then subjected to filtering in an automated Excel spreadsheet. Of each section, 8 points were selected (those lying the closest to the x- and y-axis in positive and negative, as well as x = y and x = −y functions) and stored in another sheet.

The result of the automated data processing algorithm is an Excel spreadsheet, and the data are fed to the 3D model in the Inventor software. The operations after the scanning are realized mostly automatically, thanks to appropriately written macros and scripts. Certain simple manual operations are required at this stage—these are the manual measurement of the stump length and the positioning of its digital model in the center of the coordinate system. However, most of the process of data preparation can be considered as automated. The whole process takes approximately 30–45 min (including realization of both scans and data preparation).

**Figure 5:**
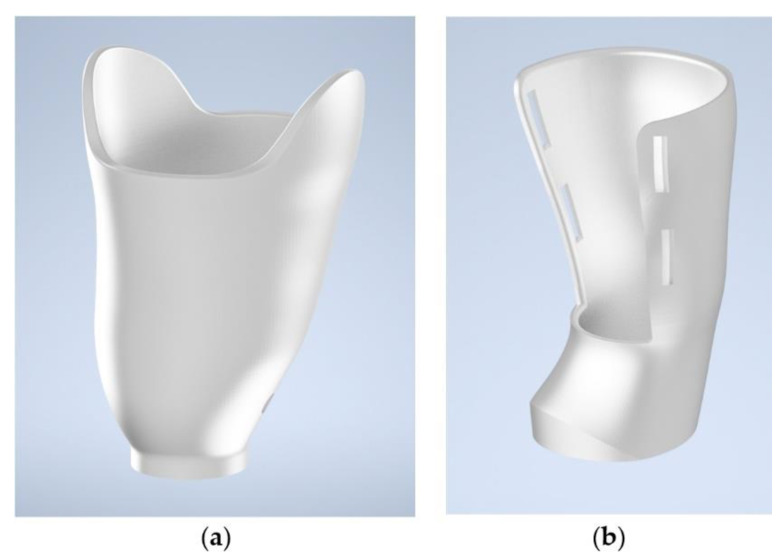
Automatically designed sockets for a customized prosthesis. (**a**) Vacuum socket (VAC) and (**b**) open socket (VEL).

On the basis of the obtained measurements, two sockets of different designs were prepared. The first one was a typical vacuum prosthetic socket, fully encircling the patient’s arm. The second one was an open socket, secured over the patient’s arm by use of Velcro straps. Both sockets have a wall thickness of 4 mm. The wall thickness was selected arbitrarily on the basis of existing, examined prosthetic sockets (commercial ones) and previous experiences of the authors, e.g., in production of orthoses [30], to make sure that the strength of the sockets would be sufficient. These two sockets were given codenames of VAC and VEL, respectively, to be used in experiments and the description of the results. Both models are presented in Figure 5. The models were exported to the STL format to be prepared for manufacturing by 3D printing.

### 3.3. Manufacturing

The automatically designed individual prosthetic sockets were manufactured with varying materials and process parameters. Destructive and nondestructive testing was performed on the obtained products. For each product, four aspects were assessed: manufacturing process stability, economical aspect (manufacturing time and total cost), accuracy (patient fit, measured surface quality), and strength (maximum force recorded at the compression test). Variability of manufacturing parameters was limited to values selected in previous studies. The changeable parameters (apart from materials) were layer thickness and infill percentage. All the sockets were manufactured in vertical orientation, selected for better accuracy and easy post-processing (no support structures).

The manual post-processing of the obtained products was limited to the simplest activities: support removal, basic manual grinding, and thermal removal of excess strings of material. Fit, accuracy, and strength were assessed after processing.

The manufacturing processes were realized with a FlashForge Creator Pro machine (Flashforge3D technology Co., LTD, Jinhua, China), with a working chamber sized 227 mm × 148 mm × 150 mm, with a dual extruder, each with a 0.4 mm diameter nozzle. This machine can be classified as a low- or medium-cost 3D printer, with an approximate purchase cost of $750. Two materials were used: polylactic acid, PLA (Spectrum Group, Pęcice, Poland), and thermoplastic polyester, TPE (Fiberlab S.A., Brzezie, Poland), in the form of 1.75 mm-diameter filaments. Material processing characteristics (based on the material supplier data) are presented in Table 1. Constant material parameters were kept during manufacturing. The temperatures and extrusion speeds were selected after the preliminary tests on simple samples, and the most suitable values recommended by the producer were selected (utilizing the most stable process, without layer disjoint, under-extrusion, or other typical errors in the FDM process). In terms of PLA, the temperature was slightly higher than is usually found in industrial practice—it was a purposeful increase aimed at deeper diffusion, better inter-layer connections, and, in consequence, a slight reduction in possible anisotropy of the mechanical properties of obtained sockets. The selected process parameters are shown in Table 1. The values of other manufacturing parameters (not shown in the table) were assumed standard as recommended by machine and material manufacturers.

Three differing strategies were formulated regarding the layer thickness and infill:

- Economic (econo)—10% infill, 0.3 mm layer;
- Accurate (accura)—10% infill, 0.1 mm layer;
- Strong (strong)—90% infill, 0.3 mm layer.

Solid, monolithic infill was avoided, as an increase in infill above 90% would not bring any substantial raise in mechanical properties due to low wall thickness, as suggested by results of previous studies; instead it would negatively influence the time and cost of manufacturing. Moreover, for the PLA material, 100% infill can easily cause part accuracy and quality issues; filament diameter must be exactly consistent. For each unique combination of process parameters (material, orientation, and strategy), 3 complete sockets were manufactured. Each socket was a single specimen subjected to strength and surface quality tests (the results were averaged), selected specimens were used for fitting tests with the patient. In total, 36 complete sockets were manufactured and tested (resulting in 2 designs, 2 materials, 3 strategies, and 3 specimens per each unique combination). Names of particular series of samples were created as a juxtaposition of design (VAC and VEL), material (PLA and TPE), and strategy (econo, accura, and strong) codenames. For example, designation of “VAC_PLA_econo” means a vacuum type of socket, made of PLA, using the econo strategy (10% infill and 0.3 mm layer).

### 3.4. Product Assessment Methodology

As mentioned above, the following aspects were assessed in the obtained products:

- Process coefficients: (a) process stability—how many process instances were stable without operator intervention on a fully functioning machine without avoidable operator error; (b) process errors—visible deformations that could disqualify the socket from use;
- Economic coefficients: manufacturing time and cost;
- Technical coefficients: strength (force at break in the bending test) and accuracy (fit/no fit, surface quality assessment, and 3D scanning of representative sockets).

In terms of process coefficients, the process was considered stable when it could be left unsupervised for the whole duration and yield a usable product. Major failures, such as product disjoint from the machine table, were considered as instabilities, while minor errors (such as visible droplets of material, stringing, unwanted holes in closing layers, etc.) were not; however, they were assessed in terms of how they could possibly affect the function or comfort of the prosthesis.

Considering the economic coefficients, real machine work time was measured, as was real material consumption. The cost was calculated similarly to how a commercial order would be priced, including the material consumption by weight, manufacturing time (calculated based on the market price of the machine, divided by the standard period of consumption of fixed capital—2 years of work on a single shift), and operator work time.

The patient fit was tested digitally and used a 3D-printed phantom of the patient’s limb. The patient also tested certain representative sockets, wearing them for several minutes—fitting and general impression were surveyed. Selected sockets representing all the materials, designs, and strategies were also subjected to acceptance of a qualified physiotherapist.

The accuracy and surface quality were also measured objectively—the sockets were 3D scanned and tested using a profilometer. The strength tests were performed as a final stage. The tests are described in more detail in next chapters.

As a final assessment of a given product, a decision of acceptance/rejection was made. The rejection criteria were as follows:

- Manufacturing stability: less than 100% process stability (no 3 successfully produced instances);
- Accuracy: more than 1 mm of average dimensional error between socket and stump, more than 50% points exceeding mean deviation of 1 mm from the nominal CAD geometry;
- Fitting: lack of fitting to the patient, unacceptable discomfort, skin irritation, too heavy, and general lack of acceptance towards a given solution;
- Mechanical properties: lack of strength (less than 1000 N at the moment of product failure);
- Cost: exceeding a rough estimate of 100 USD for a complete prosthesis assumed as a threshold; this is a result of a survey made among several potential patients, where a question of “how much are you willing to pay?” was asked;
- Time: too long manufacturing time (longer than a single work shift—8 h).

The surface roughness tests were not taken into account as the rejection criteria and their threshold values were not set for this study, due to the low experience of authors with assessing the correlation of these test results with the general usability of a 3D-printed medical product. However, the general purpose of surface roughness testing is to create an objective, measurable criterion of surface quality, separate from subjective assessment by the patients, so it was assumed that a certain value of roughness will be determined as a threshold value, based on the comparison of measured values to the patient’s assessment.

### 3.5. Strength Testing Procedure

**Figure 6:**
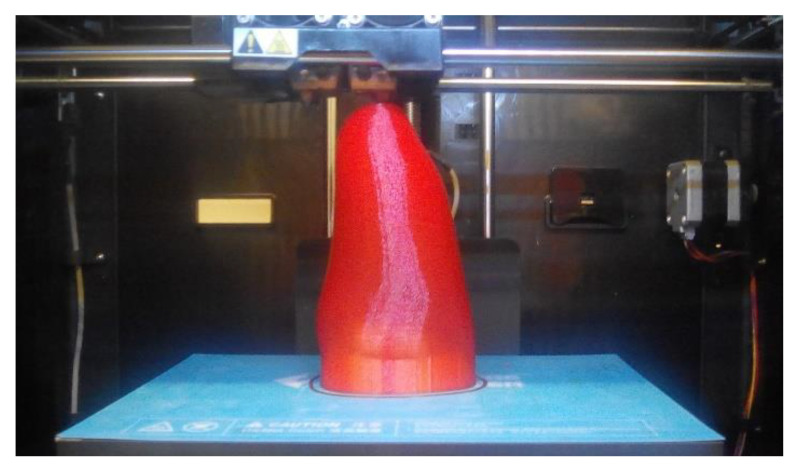
Manufacturing of elastic stump shell for the strength test.

The strength testing of the manufactured upper-limb prosthetic sockets consisted of a destructive quasi-compression test of the whole socket. To simulate load with a patient’s stump inside, a two-material phantom was manufactured. Based on the 3D scan, an outside shell and a core in the shape of a conical rod were modeled. The shell was made of TPE (elastic thermoplastic polyester) (Fiberlab S.A., Brzezie, Poland) material, infill 10%, and layer height 0.3 mm (Figure 6), and the core was made of PLA (Spectrum Group, Pęcice, Poland), infill 30%, layer height 0.4 mm, and 4 contours. The phantom was placed inside every tested socket. The strength tests were performed with the universal strength testing machine Sunpoc WDW-5D-HS (Sunpoc, Guiyang, China). The course of the experiment was developed based on ISO 604: 2002 standard [35]. The load was applied along the stump main axis, from the upper side. This type of load was selected as this is the area where the greatest forces may occur in everyday life—in the case of stretching or bending the stump under heavy load it will usually leave (slip off) the socket. It is also quite common to use this type of prosthesis as a support for the patient’s own body weight. The compression also makes it easier to draw potential comparisons with lower-limb sockets.

**Figure 7:**
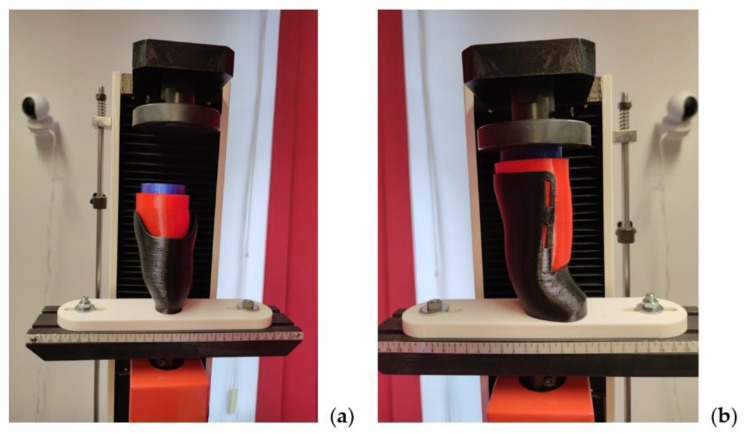
Two-material stump phantom used in the strength testing of the VAC (**a**) and VEL (**b**) sockets.

Special-shaped support was designed and manufactured out of PLA material (with monolithic infill of 90% to avoid deformation during the test) and placed and screwed to the test machine’s rail. During loading, sockets were not additionally fixed—the supports significantly limited the freedom of movement of the product. In the VEL sockets, the eponymous Velcro straps were used to secure the stump, as they would in a real-life scenario. Velcro straps of 20 mm width and 1.5 mm thickness were used. In the VAC sockets, no additional equipment was used. The whole set for both sockets is presented in Figure 7.

The result of each test is a course of a load–displacement diagram, obtained from the used strength testing machine. The test was carried out until one of the following conditions occurred:

- Failure of a given socket (visible and/or audible fracture, visible, continuous plastic deformation);
- The load value of 4000 N was exceeded;
- The stump phantom slipped off a given socket.

As a threshold of positive evaluation of sockets that were actually damaged, a load of 1000 N was assumed (representing a static compression using an item of ~100 kg mass). There are no widely available standards for conducting such tests on 3D-printed sockets. However, the ISO 10328 standard (testing of lower-limb sockets [36]) was looked into as a partial reference—using this standard, loads between 60 and 125 kg are assumed for static tests of lower-limb prostheses [37]. Therefore, the force condition of 1000 N was assumed, also based on discussion in the project team, with mechanical and biomedical engineers, and with expert participation of prosthetic technicians and physiotherapists, who stated that in practice, such sockets do not need to bear higher loads.

### 3.6. Accuracy Testing Procedure

The accuracy of the manufactured sockets was tested in a non-destructive procedure–3D optical scanning by a professional, industrial-grade 3D scanner.

In order to verify the geometry, the prosthetic sockets were measured with a structured light 3D scanner. For this purpose, the GOM Atos Compact Scan 5M scanner was used, equipped with a measuring field size of 300 mm × 230 mm. Non-coded points (round markers of 3 mm diameter) were used to connect individual scans. They were placed directly on the measured objects. Three-dimensional scanning made it possible to perform a non-contact measurement and recreate digital representations of prosthetic sockets. The result of a single scan was a triangular mesh saved in the STL format. Thanks to the use of a 3D scanner with blue light, there was no need to use chalk powder, which could affect the strength tests carried out in the next stage. A total of 4 socket variations were selected for the measurement—both designs and both materials (VEL/PLA, VEL/TPE, VAC/PLA and VAC/TPE). All the selected sockets were produced in the econo strategy, the parameters of which may lead to the largest errors in mapping the socket geometry.

The most important parameter of the prosthetic socket is its fit to the stump, therefore only the part of the socket that has direct contact with the stump was used for the inspection analysis. The homing in the common coordinate system was done by a best-fit fit between the stump scan and the part inside the socket scan. The results of accuracy testing include fit deviation for individual sockets and colorful deviation maps for all the tested sockets.

### 3.7. Surface Quality Testing Procedure

The surface quality of the manufactured sockets was tested in a non-destructive procedure—by use of a certified roughness tester. All the sockets were measured with the PowerSurf ART-300 Surface Roughness Tester (PowerTech s.c., Grojec, Poland). The device is compatible with appropriate EN ISO 4287 and EN ISO 4288 standards—it has a diamond measurement head of 5 ± 1 µm radius, and its measuring load does not exceed 0.5 N. The roughness measurement was made in 3 places on the inner part of the sockets (all measurement points were at the same height of a socket and were distributed around its circumference). The measurements were made along a constant distance of 2.5 mm, with a velocity of 1 mm/s. Five repetitions were performed at each location.

**Figure 8:**
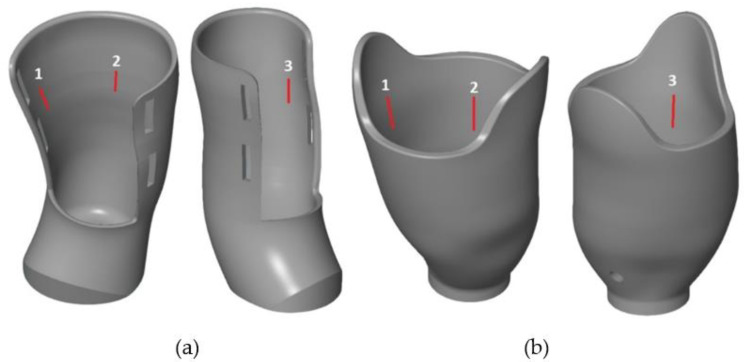
Places where the roughness measurement was carried out, socket type: (**a**) VEL and (**b**) VAC.

The measurement locations for the VAC and VEL sockets are shown in Figure 8. During the measurement, the socket was attached to the handle with its lower part using a screw, which guaranteed stability during the measurement, while the profilometer was mounted on a dedicated precision tripod. The result of the roughness measurement were the parameters of R_a_, R_z_, and R_q_. They were later subjected to subjective assessment made by the patient and physiotherapist.

## 4. Results

### 4.1. Manufacturing Results

The manufacturing was realized according to the plan and obtained a complete set of prosthetic sockets. No major process stability problems occurred—all the processes were realized from the beginning till the end. Of the several problems that were noticed, most were a result of operator error (cooling turned on too late and/or partially clogged extrusion head). In the case of an unstable process when the instability source was detected as human error, the process was repeated (it happened just once during the whole study). All the other problems were minor, which means they did not cause failure in the manufacturing of a given part, but they influenced the visual appearance and subjective quality of obtained sockets. Therefore, the processes were assumed as stable—36 sockets were obtained successfully. The minor problems observed in the studies are listed below.

**Figure 9:**
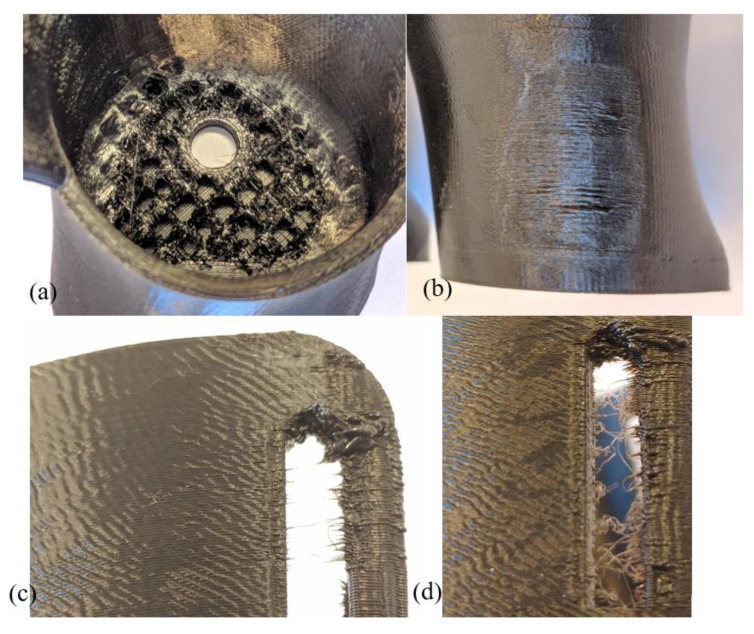
Minor manufacturing errors observed in the studies: (**a**) holes—TPE, (**b**) deformations—PLA, (**c**) deformations—TPE, and (**d**) stringing—TPE.

1. Manufacturing of TPE with a layer thickness of 0.1 mm causes the upper (closing) layers at the bottom of the socket to produce incorrectly, resulting in holes in the surface of the size corresponding to the distance between the threads of the interior fill. This effect is much more visible on the VEL socket as it has a much larger flat surface at the bottom of its cavity (Figure 9a). For just one sample, holes in the surface also appeared on the bottom surface (adhering to the table).
2. Using PLA, only for a layer thickness of 0.1 mm was it found necessary to turn on nozzle cooling (fan turned on). Thermal deformations caused by local overheating of the product were visible especially on the VEL sockets, at a height of about 10 mm from the table level (Figure 9b). In the part where the socket expands, there was no case of thermal deformation.
3. The TPE material does not require nozzle cooling. However, thermal deformations are also visible on sockets made of this material, in the VEL version. They appear within the Velcro fastening area, on the side of the wall with a smaller cross-sectional area. The machine applies the current layer at this point and immediately starts applying the contour to the next layer on a very small surface. As a result of the accumulation of too much heat, the applied material deforms in a way that is visible to the naked eye (Figure 9c). The joint between layers, though, is strong enough to allow Velcro fastening.
4. In the case of TPE orthoses, a stringing effect appears in the Velcro openings (Figure 9d).

**Figure 10:**
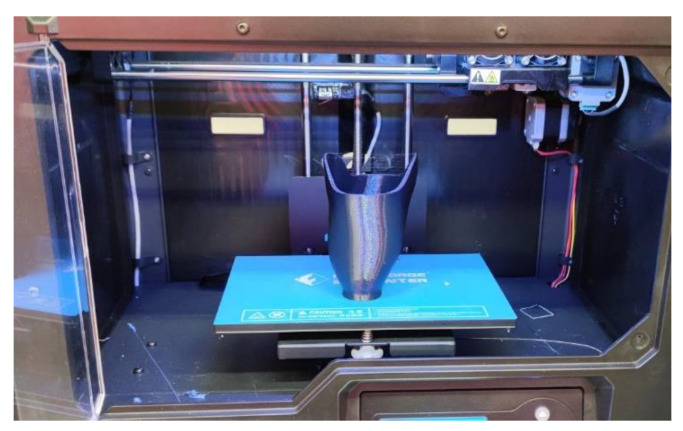
Vacuum prosthetic socket manufactured out of PLA (right before removal from the machine), accura strategy.

Figure 9 presents the examples of all the above-mentioned problems. They were decided to be minor and usually easy to deal with, by process parameter correction, avoiding operator errors (such as not turning on cooling), and post-processing. Additionally, almost no problems were observed in the VAC sockets (Figure 10), mostly due to their geometry, forming a relatively thick conical shell without any larger openings, thus making them more stable and less prone to deformations, creation of unwanted holes, and threading.

The manufactured sockets were generally very lightweight. Sockets made of PLA, which is more dense than TPE, weighed between 49 to 80 g, depending on the design and strategy (obviously, strong strategy yielded the heaviest sockets). The TPE sockets weighed between 44 and 55 g. The only two influential factors here are the infill (strong versus two other strategies) and material density, layer thickness having no statistical impact on the total weight. The two designs are very similar in terms of volume, as such the material consumption is also very similar between them.

### 4.2. Economical Coefficients of Manufactured Sockets

**Figure 11:**
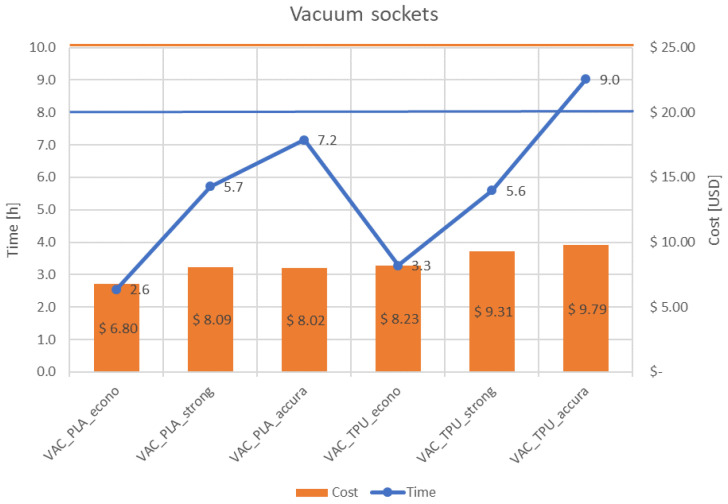
Cost and time of manufacturing—VAC socket.

**Figure 12:**
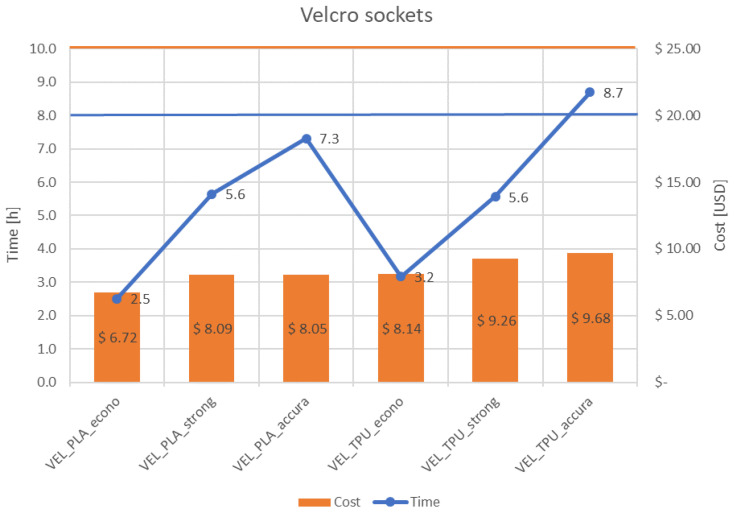
Cost and time of manufacturing—VEL socket.

Figure 11 and Figure 12 present juxtaposition of costs and times of manufacturing of the sockets, separately for each design. An acceptable price was set at 25 USD—it was mentioned before that the patients are willing to pay no more than 100 USD for a complete prosthesis, while it can be divulged (from the authors’ experience and an analysis of prices of commercial products and their spare parts) that the cost of a socket of a non-electrical prosthesis is approximately ¼ of its total price. Therefore, the acceptable price was divided by 4 and this limit was set as a threshold of rejection of the sockets. An acceptable time of manufacturing the socket was assumed as 8 h, which is a single work shift. This is enough time (in perfect conditions) to supply a patient with a full prosthesis on the second day after scanning, or even the same day, if all the parts are produced at the same time using several 3D printers.

As shown in Figure 11 and Figure 12, nearly all the proposed process parameter combinations make the production of affordable prostheses possible. The one combination that was rejected due to too long manufacturing time was of TPE material and the accura strategy (0.1 mm layer thickness), which was unacceptable for both designs due to it exceeding 8 h production time.

The considered prosthetic sockets can be manufactured much cheaper than the equivalent commercially made prostheses. This is due to the complete removal of design costs, which could be very high in these types of individualized prostheses. In the proposed approach, it could be neglected, as the prosthesis is designed automatically in less than an hour since the initial scan of the patient’s limb, without the engagement of a specialist (engineer). The proposed method of automated design allows only the manufacturing costs to be considered, which are relatively small, using low-cost materials and 3D printers. This is a large opportunity, unknown before for pediatric patients all over the world, and it should be explored in further studies.

### 4.3. Accuracy Test Results

The results of best fit—the mean deviation—between the 3D scans of the selected sockets and the scan of the patient’s stump are presented in Table 2.

The highest deviation is for the vacuum socket made of elastic TPE material. The lowest is for the Velcro-fastened socket made of more rigid PLA. All the deviations are less than 1 mm and near, or slightly exceeding, the layer thickness in the econo strategy. In view of such results of the “worse” strategy, it was decided not to 3D scan the sockets manufactured in the accura strategy, as the results of the econo strategy are fully acceptable (below an assumed limit of 1 mm).

**Figure 13:**
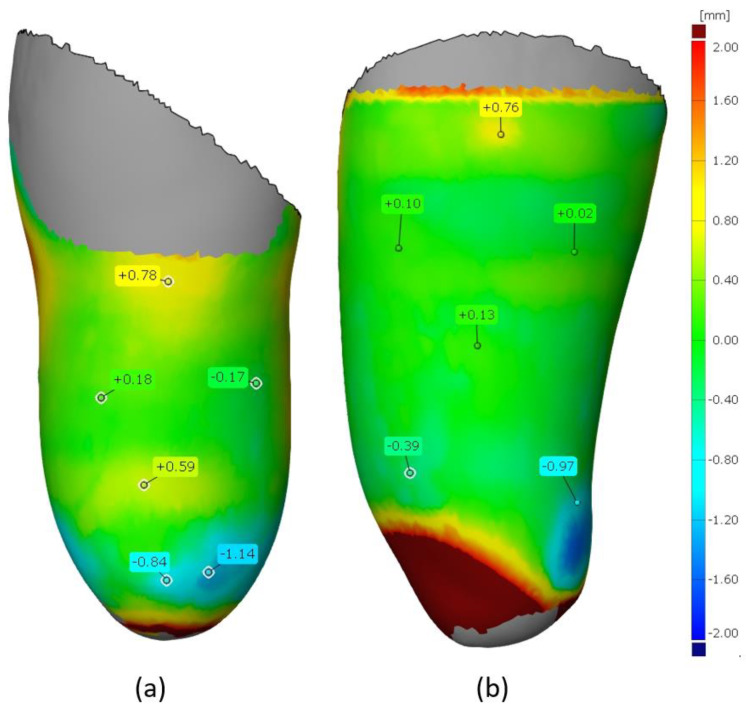
Deviation map for the sockets of PLA. Type: (**a**) VAC and (**b**) VEL.

**Figure 14:**
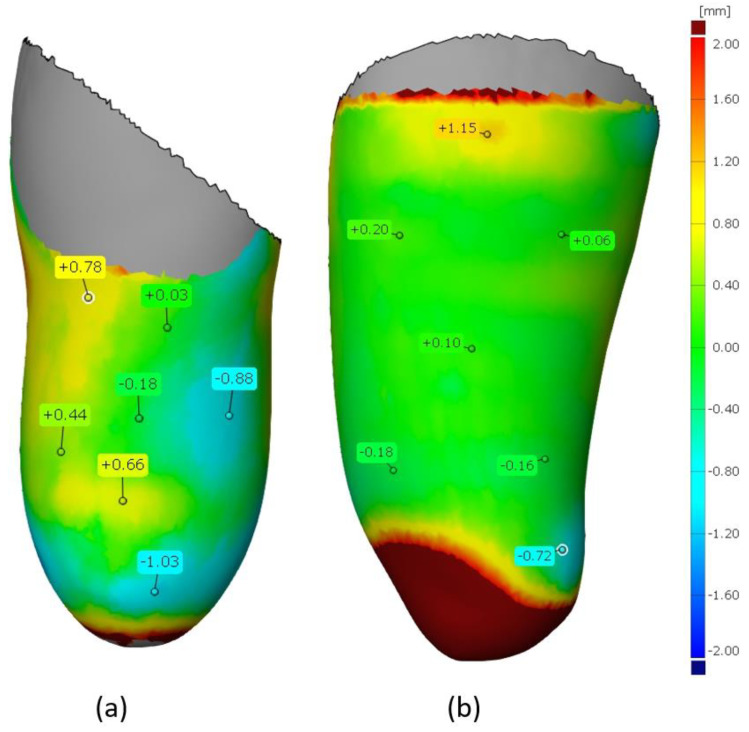
Deviation map for the sockets of TPE. Type: (**a**) VAC and (**b**) VEL.

**Figure 15:**
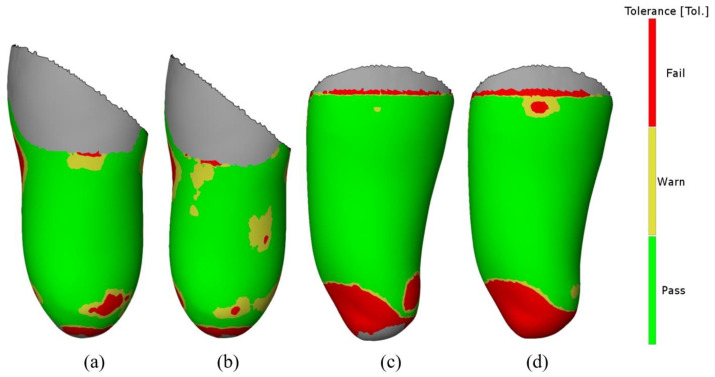
Deviation map regarding the assumed 1 mm tolerance: (**a**) VAC PLA, (**b**) VAC TPE, (**c**) VEL PLA, and (**d**) VEL TPE.

The results of the comparison of the inner parts of the sockets are shown in Figure 13 (for the VAC type) and Figure 14 (for the VEL type), as mentioned above—considering only the “worse” strategy (0.3 mm layer thickness). It can be noticed that in both types of sockets, lower deviation values are found for the ones made of PLA material. This may be related to the higher processing shrinkage of TPE material, which affects the deformation of a given socket geometry. Figure 15 shows the analysis for a tolerance of ±1 mm. In all measured objects, 90% of all control points fell within this range. Therefore, no socket was rejected due to low accuracy criteria (mentioned in Section 3.4).

### 4.4. Surface Quality Test Results

**Figure 16:**
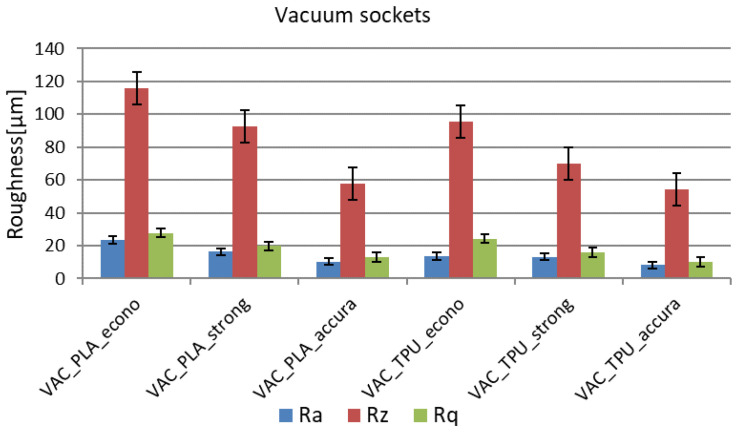
Roughness measurement results for the vacuum sockets.

**Figure 17:**
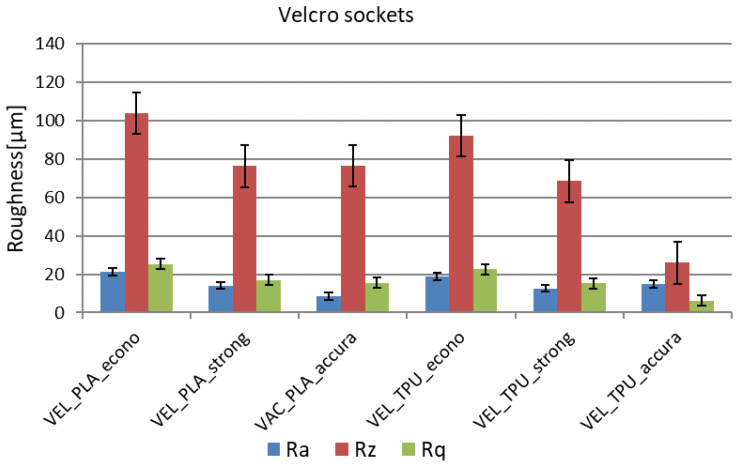
Roughness measurement results for the Velcro-fastened sockets.

On the basis of measurements carried out with a surface roughness meter, three parameters describing the roughness of the sockets were determined: R_a_, R_z_, and R_q_. The values for these parameters were calculated as the arithmetic mean and standard deviation of all measurements carried out for a given variant of the socket. The results with the division into VAC and VEL sockets are presented in Table 3. It can be noticed that the greatest influence on the surface roughness was exerted by the thickness of the layer, which has the greatest impact on the staircase effect in products manufactured by the FDM method. The sockets produced in the accura strategy were characterized by the lowest roughness and the highest repeatability of roughness parameters. According to the ISO 1302: 2004 standard, the sockets made in this strategy can be classified into the third roughness class, while the sockets made in the econo and strong strategies can be classified into the second class. The roughness measurement results, in the form of graphs, are shown in Figure 16 and Figure 17.

General observations regarding the measured surface roughness are as follows:

1. The vacuum sockets are generally more rough, despite very similar locations selected for tests in both designs (inner surface of VEL and VAC sockets is very similar in shape—both are fit to the same patient and designed on the basis of the same data).
2. The TPE material was measured as being less rough than PLA in corresponding strategies with the same layer thicknesses, for both designs and all the strategies.
3. The difference between the worst and the best socket in terms of roughness, for a given design, is threefold in the case of the R_a_ coefficient and sevenfold in the case of the R_z_ coefficient.
4. There are significant differences between the econo strategy and the strong strategy, despite using the same layer thickness (which should be the most important parameter here).
5. The best strategy, as anticipated, is the accura strategy, with the lowest layer thickness, greatly influencing the obtained results.

### 4.5. Fitting and Patient Assessment Results

In terms of fitting tests, all three tests allowed positive results to be obtained. The virtual (digital) testing results found no collisions, with the deviations calculated and shown in the previously shown Table 2. The testing on the stump phantom also did not allow any particular errors or deviations to be found—the sockets were fitting without any noticeable problems. They were also able to be properly secured—the vacuum socket did not fall off the stump phantom when held vertically (thus proving the fitting and stability); the Velcro socket was fastened on the phantom without any problems.

**Figure 18:**
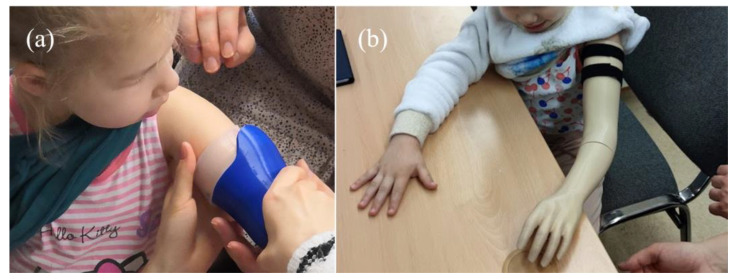
Testing of the sockets with the patient: (**a**) VAC socket and (**b**) VEL socket (in assembly with a complete prosthesis).

The tests with the patient were done as the last stage, after manufacturing the whole prosthesis. For psychological reasons, as suggested by physiotherapists, it was recommended not to fit on only the sockets, but rather full prostheses. Photographs from the testing procedure are shown in Figure 18. The general observations are listed below.

1. The patient, as well as the parent and the consulted physiotherapist, could not indicate any difference between the sockets made in the accura and econo/strong strategies in terms of fitting to the stump, except the VEL socket made of PLA. The PLA socket made with 0.3 mm layer thickness was indicated as skin irritating.
2. The VAC socket was assessed as heavy and bulky, especially the one manufactured in the strong strategy, as well as difficult to install and physically uncomfortable due to full enclosing of the arm, regardless of the material, although the TPE was received slightly better due to its flexibility. The VEL socket, due to its open geometry, was better received and assessed by the patient, also regardless of the material.
3. The VEL sockets, although fitting properly, were found to be slightly too loose, mostly due to relative shortness of patient’s stump (and lack of proper surface area to which the socket could adhere). This was more of a problem in PLA sockets, which are more rigid (less prone to fastening by Velcro straps).

### 4.6. Strength Testing Results

**Figure 19:**
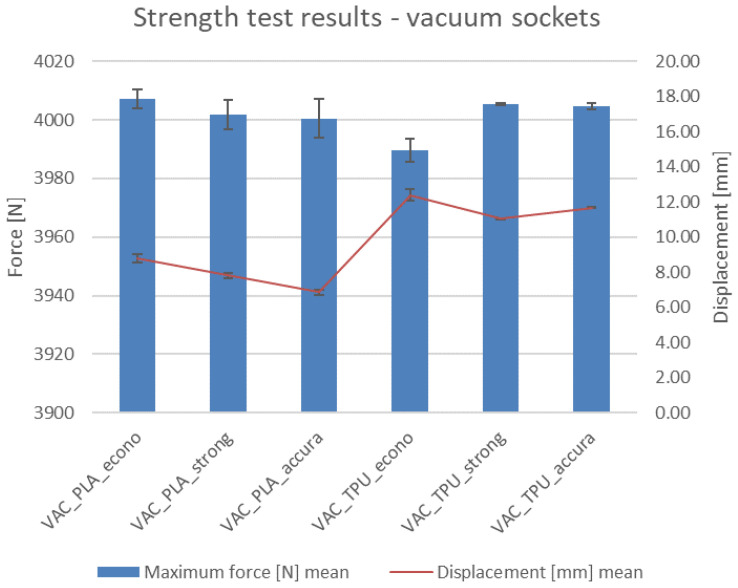
Strength test results for the vacuum sockets.

**Figure 20:**
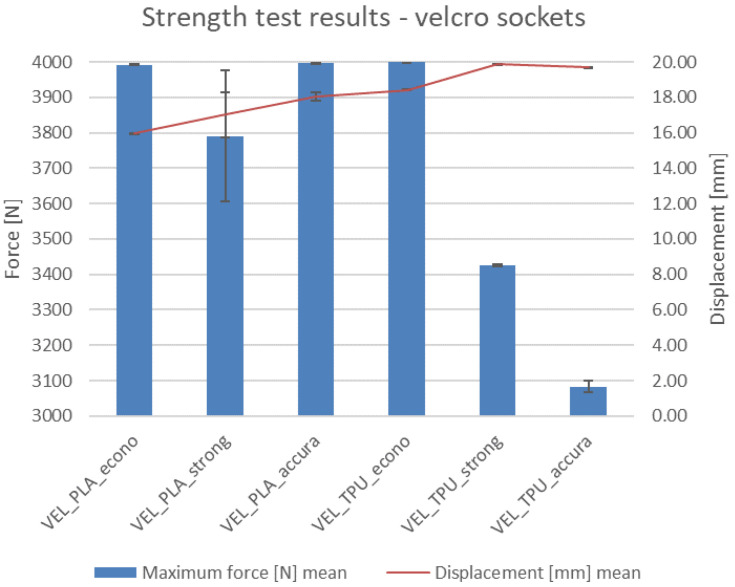
Strength test results for the Velcro sockets.

Results of the strength tests—maximal recorded force, elongation, and result of a given test—are presented in Table 4. Visible results of the tests (deformations and fractures) are shown in Figure 19 for the VAC sockets and Figure 20 for the VEL sockets. The values of force in the diagrams were appropriately scaled to visualize the differences, so the reader must pay attention to the strength axis values.

As mentioned in the methodology, the test was conducted until reaching 4 kN of force or until fracture and/or visible deformation occurred. An additional condition was the socket slipping off the stump without any sign of failure (possible only for the VEL sockets due to their semi-open geometry). This occurred for all the samples in group 11 and 12, which are Velcro sockets made of TPE, produced in the strong and accura strategies. This did not happen to the sockets manufactured using the econo strategy.

**Figure 21:**
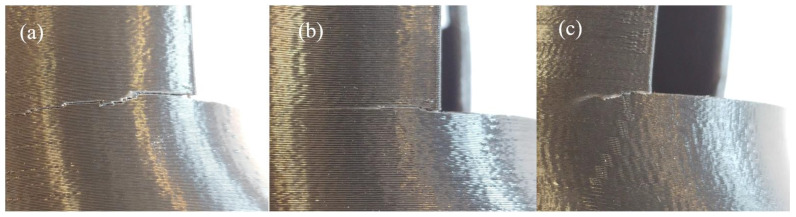
Fractures of tested VEL sockets of PLA. (**a**) Econo: large, multiple layers. (**b**) Strong: medium, single layer. (**c**) Accura: small, multiple layers.

The main, visible results of the tests are presented in photographs in Figure 21 (they refer to the only type of sockets with visible failures—VEL-PLA). The main observations are summarized below:

1. The VAC sockets were not damaged in any test, and no visible plastic deformation was observed—they could probably withstand much higher compressive force.
2. Velcro sockets made of PLA all failed regardless of the manufacturing strategy. All the fractures were located near the transition of the socket to the flat surface. This is a clear notch on which the stress concentration was carried by the stump as it deformed and “flowed” out of a socket.
3. No specimen made of TPE was either broken or delaminated. Only the Velcro sockets suffered damage (plastic deformation) of the closing layers inside the socket. It happened under the pressure of the stump. On the TPE samples manufactured with the accura strategy, there was a plastic deformation at the bottom of the socket. However, a pressure of approximately 400 kg on the bottom of the socket will not occur in a typical usage scenario, for it to be of any practical concern.
4. As anticipated, the displacement values are generally lower for the PLA material (as it is more rigid). The difference is much more prominent for the vacuum sockets—it is 40% between the respective sockets made in the econo and strong strategies and 70% for the accura strategy. For the Velcro sockets, the difference is 15% for the econo and strong strategies and 9% for the accura strategy between respective sockets made of the two materials. The maximal recorded value of displacement for all the sockets is nearly 2 cm (for the VEL_TPE_strong socket series, similar values were observed for the accura strategy).
5. In terms of the relationship of manufacturing strategy with strength and displacement, it was found that such a relationship exists. The sockets made in the econo strategy (high layer thickness and low infill) presented the highest displacement for both materials in the vacuum sockets, while in the Velcro sockets it was the opposite. The strong strategy did not bring a considerable improvement in obtained results over the econo strategy—in the Velcro sockets made of PLA (which is the only group in which actual failure occurred), the strong strategy yielded the lowest recorded strength values of all three strategies, with the accura strategy being the best one in terms of maximal force at break.
6. Fracture types in VEL sockets were different, depending on the manufacturing strategy. The most evident fractures were visible for the econo strategy (Figure 21a)—they went through many layers. In the strong strategy the fractures were smaller (Figure 21b) and going in line with a single layer. In the accura strategy, the fractures went through multiple layers, but were small (Figure 21c).
7. In the series no. 8 (VEL_PLA_strong) one specimen fractured at a value of approximately 3400 N, with deformation of 19 mm. This heavily influenced the mean and standard deviation values, as another two specimens fractured rather similarly, at approx. 4000 N and with 15 mm of deformation. This could be a result of a cumulation of internal stresses due to internal structural error, caused by monolithic infill (a phenomenon known to the authors from many previous studies).
8. The stump phantom withstood all the strength tests, despite the heavily deformed TPE part at each test. However, the deformations were of an elastic character—the geometry always returned to its original shape after a test.
9. It was not possible to study the transverse loads of the VEL sockets as the stump was leaving the funnel under the influence of the applied force. The question is how big the force securing the stump to the socket is, and whether in real use the stump will fall out of the socket sooner than the failure occurs.
10. Velcro straps used in the tests (width 20 mm and thickness 1.5 mm) do not show any signs of deterioration, despite the repeated use of the same ones in strength tests. This means that they could be much thinner, but it would probably affect the comfort of using the socket.

In general, no socket failed or deformed below the set threshold of 1000 N of maximal compressive force. Most of the tested sockets reached values well above 3500 N without signs of failure. It means that their geometry could be topologically optimized for less volume and weight and better comfort of the patient—in practice, with the assumed design and manufacturing parameters, no damage should occur in any situation of day-to-day use, even assuming dynamic movement of a prosthesis user (bicycle or scooter riding, jumping, etc.).

### 4.7. Final Assessment

Results of the final assessment are presented in Table 5. The fulfillment of specific criteria are marked as “+”, meaning positive, “–“, meaning negative, and “0”, meaning neutral (neither positive nor negative, or in between).

The following variants of the manufactured sockets are the ones that were not rejected, considering all the assessment criteria:

1. VEL socket made of PLA, manufactured in the accura strategy.
2. VAC socket made of TPE, manufactured in the econo strategy.
3. VEL socket made of TPE, manufactured in the econo and strong strategies.

Taking into account only the objective, measurable qualities of the sockets manufactured by 3D printing of PLA and TPE materials, most of the produced variants were acceptable. In terms of purely technical coefficients (manufacturing stability, accuracy, and strength), every single socket fulfilled them all, with the VEL sockets of the PLA material receiving worse results in strength tests (all sockets failed eventually, as opposed to the TPE sockets), but ones that were still acceptable. Adding economical coefficients to the mix, the econo strategy is acceptable in all cases, as is the strong, but the accura is not for the TPE material; this is because of the long manufacturing time, which exceeds 8 hours.

However, the most severe rejection criterion is fitting and final acceptance by the patient. Only selected variants of the prosthetic sockets were accepted, these were Velcro sockets of TPE—regardless of strategy, PLA—only in the accura strategy (less skin irritating), and partially vacuum sockets of TPE—except the strong strategy, which made the socket too heavy. It means that even objectively well-made medical products may still be rejected by their recipients, especially when pediatric patients are considered (when reasons of rejection could be purely subjective). This needs further exploration in a larger group of patients, to determine correlation and causation between certain values of technical coefficients and acceptance ratios of patients.

## 5. Discussion

The main goal of the performed experiments was to establish a set of parameters allowing prosthetic sockets to be manufactured by means of 3D printing, meeting all the technical and economic criteria. The results allow us to state that it is completely feasible to manufacture fully functional prosthetic sockets using standard 3D printing technologies and readily available materials. It was also proven that it is possible to supply a patient with a complete, individualized prosthesis after 24 h from the first contact (3D scanning), and the material processing via fused deposition modeling technology is not a hindrance in this process.

The TPE material was found to be objectively more suited to the task than the popular PLA material. It is slightly less dense (2% difference), thus enabling sockets of lower weight to be obtained. It is considerably stronger and also less rough. However, its slightly higher processing shrinkage caused the obtained sockets to be less dimensionally accurate, although fully acceptable from the practical point of view. The TPE may be problematic in terms of post-processing, as it is very difficult to improve its surface quality by mechanical or chemical means. Thus, the accura strategy would be the one to recommend, but TPE also takes considerably longer to process by 3D printing than the other materials (including PLA and other popular materials, such as ABS or PET-G). As such, prosthetic sockets made of TPE and manufactured in the accura strategy are less feasible in terms of the economics and logistics of the prosthesis production process (it would take more than one day to assemble the full prosthesis, even using multiple 3D printers at once).

It is also noteworthy that the 3D-printed TPE material has a higher general acceptance rate than the PLA material, although a study on a larger group of patients must be done to confirm this fact, aside from the case study presented in this paper. Additionally, the socket with the worst surface roughness was also correctly identified by the patient, parent, and physiotherapist—it was the VEL socket made of PLA in the econo strategy (the VAC PLA econo socket was objectively worse, although similar, but it was rejected by the patient for other reasons). On the basis of the obtained results, the authors propose to set the criterion of roughness of prosthetic sockets as R_a_ < 15 µm. However, further studies are needed with the patients to test the effects of 3D-printed sockets on human skin due to its considerable roughness, especially with pediatric patients.

The sockets made of PLA material have good accuracy and acceptable strength, with very good economic coefficients. The PLA should be manufactured with lower values of layer thickness to avoid higher roughness, which leads to discomfort during use by a given patient. Alternatively, it should be subjected to grinding or lining with an elastic, skin-pleasant material (e.g., a foam). Both approaches are currently being tested by the authors, with the foam lining approach obtaining better results in terms of patient acceptance. Still, manufacturing the socket in the econo strategy generates some practical issues, for instance with clothes (the outer surface is also rough and could interfere with the fabric of patients’ clothing).

The proposed strategies of manufacturing manifested in differences in obtained test values. In terms of accuracy and roughness, the trends were compatible with pre-study assumptions. However, in terms of strength, no anticipated increase was obtained in the case of the “strong” strategy. As the strategy does not allow any significant improvement over the two other strategies to be obtained, it was discarded from future considerations. The remaining two strategies both allow the obtaining of usable sockets. The user of the AutoMedPrint system will be given a choice between better accuracy, roughness, and looks with a longer delivery time (accura strategy), or a shorter delivery time with worse roughness and appearance of the prosthesis (econo strategy).

**Figure 22:**
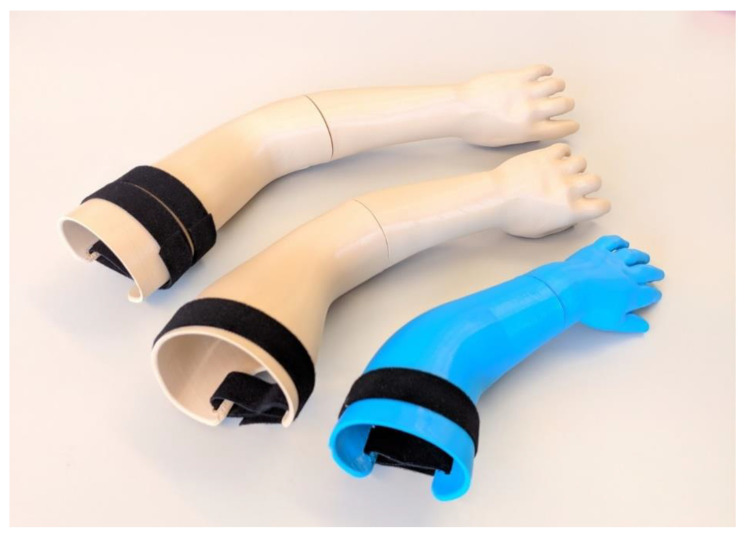
Prostheses made for pediatric patients and put to use on the basis of the study results.

Considering the design, it clearly can be improved for better results. The vacuum sockets, although of the same wall thickness and roughly the same mass as the Velcro sockets, were not damaged in the strength tests. This mean that their geometry could be topologically optimized for less material use and possibly better reception by the patients. Several various designs, including the openwork ones (compression/release-stabilized CRS sockets), are currently under consideration and further study. The authors also developed a new method of FEA-driven optimization of orthopedic equipment, described in previous studies [27]. This method could be potentially automated, in order to facilitate selection of appropriate wall thickness for a given patient and application. In terms of manufacturing, it can be assumed that in general, given the set of applied process parameters, acceptable stability was achieved—the process does not require constant supervision by an operator. It must be remembered that geometry of the sockets is individualized and different every single time the system is used for designing a new prosthesis. However, in the authors’ opinion, it is highly improbable that any variation in socket geometry could cause the manufacturing process to become unstable in the way that would not allow a functioning, acceptable prosthesis to be obtained. This set of manufacturing parameters for the two materials and designs was further tested on a group of sockets manufactured for another three patients, aged 4, 3, and 1. The results (finished prostheses) for the VEL design and TPE materials are presented in Figure 22. All the prostheses were accepted by the patients and physiotherapists and have been put to use.

## 6. Conclusions

All research was conducted as planned and the results are mostly compliant with initial expectations of the researchers. The results of the studies have proved that it is possible to obtain a usable and cheap 3D-printed hand prosthesis in less than one workday, from measurement to a ready product, with minimal involvement of a human operator and minimal competences required to perform the whole process. The proposed approach for manufacturing prosthetic supplies is reliable to use in hospitals, doctors’ offices, and other medical facilities, with no full-time involvement of an engineer or technician. The 3D printing itself may be realized as an external service.

The study results and conclusions were considered in the next phase of the project, building the interface for the system’s user (with appropriate selection of material and manufacturing strategy). Future studies planned on the scope of the project are experimental tests of more materials (including nylon) and clinical tests, with the engagement of more patients (children and adults). So far, the prostheses were made for 5 patients due to restrictions enforced by COVID pandemics, but the authors expect the number to be growing in the near future.

## Figures and Tables

**Table 1:** Characteristics of materials used and FDM process parameters.

No.	Name	Properties	Process Parameters
1	PLA—polylactic acid	Density: 1.24 g/cm^3^ Extrusion temperature range: 185–230 °C	Extrusion temperature: 220 °C Build platform temperature: 50 °C Extrusion velocity: 60 mm/s Number of contours: 2 Number of closing/opening layers: 3/3
2	TPE—thermoplastic polyester (Fiberflex 40D)	Density: 1.21 g/cm^3^ Extrusion temperature range: 200–240 °C Declared Shore scale hardness: 40D	Extrusion temperature: 235 °C Build platform temperature: 60 °C Extrusion velocity: 40 mm/s Number of contours: 2 Number of closing/opening layers: 3/3

**Table 2:** Deviation of fit between sockets and the stump.

Type	Material	Manufacturing Strategy	Deviation (mm)
VAC	PLA	econo	0.324
VAC	TPE	econo	0.398
VEL	PLA	econo	0.296
VEL	TPE	econo	0.396

**Table 3:** Results of roughness testing of the manufactured sockets.

No.	Socket Type	R_a_ (µm)		R_z_ (µm)		R_q_ (µm)	
		Mean	Std. Dev.	Mean	Std. Dev.	Mean	Std. Dev.
1	VAC PLA econo	23.39	4.29	115.91	13.88	27.81	4.54
2	VAC PLA strong	16.28	1.58	92.66	9.45	19.78	1.91
3	VAC PLA accura	10.39	1.03	57.63	18.72	12.96	1.54
4	VAC TPE econo	13.63	2.93	95.27	15.44	24.24	3.64
5	VAC TPE strong	13.22	1.76	69.83	9.82	15.94	2.04
6	VAC TPE accura	8.24	1.57	54.27	11.91	10.13	2.28
7	VEL PLA econo	21.30	1.06	104.00	5.76	25.22	1.17
8	VEL PLA strong	14.13	1.60	76.30	6.55	17.04	1.78
9	VEL PLA accura	8.37	0.48	76.48	2.17	15.43	0.55
10	VEL TPE econo	18.82	0.90	92.16	6.98	22.58	1.15
11	VEL TPE strong	12.62	0.73	68.47	6.14	15.24	0.90
12	VEL TPE accura	15.03	1.02	25.90	5.94	6.28	2.40

**Table 4:** Results of strength testing of the manufactured sockets.

No.	Series	Maximum Force (N) Mean	Displacement (mm) Mean	Failure/Deformation	Damage Type
1	VAC_PLA_econo	4007	8.81	No	None
2	VAC_PLA_strong	4002	7.81	No	None
3	VAC_PLA_accura	4000	6.85	No	None
4	VAC_TPE_econo	3989	12.39	No	None
5	VAC_TPE_strong	4005	11.04	No	None
6	VAC_TPE_accura	4005	11.67	No	None
7	VEL_PLA_econo	3992	15.97	Yes	Fracture
8	VEL_PLA_strong	3790	17.02	Yes	Fracture
9	VEL_PLA_accura	3997	18.07	Yes	Fracture
10	VEL_TPE_econo	3999	18.43	No	None
11	VEL_TPE_strong	3426 *	19.88	No	None
12	VEL_TPE_accura	3083 *	19.69	Yes	Deformation

*, Stump slipped off the socket (test stopped).

**Table 5:** Final assessment of the produced prosthetic sockets. Strategies: E—econo, A—accura, and S—strong; if no strategy indicated, assessment applies to all of them.

No.	Criterion	VAC PLA	VEL PLA	VAC TPE	VEL TPE
1	Manufacturing	+	+ *	+	+ *
2	Accuracy	+	+	+	+
3	Fit and acceptance	– **	E/S: – ** A: +	E/A: 0 S: – **	+
4	Strength	+	+	+	+
5	Cost	+	+	+	+
6	Time	+	+	E/S: + A: –	E/S: + A: –

*, Minor errors. **, Unaccepted by the patient.

## Data Availability

The data presented in this study are available on request from the corresponding author. The data, apart from those available explicitly in the paper, are not publicly available due to privacy of the patient whose anthropometric measures were used in the paper.
